# Detrimental effects of albuterol on airway responsiveness requires airway inflammation and is independent of β-receptor affinity in murine models of asthma

**DOI:** 10.1186/1465-9921-12-27

**Published:** 2011-03-07

**Authors:** Lennart KA Lundblad, Lisa M Rinaldi, Matthew E Poynter, Erik P Riesenfeld, Min Wu, Steven Aimi, Leesa M Barone, Jason HT Bates, Charles G Irvin

**Affiliations:** 1 Vermont Lung Center, Department of Medicine, University of Vermont, 149 Beaumont Ave, Burlington, VT 05401, USA; 2 131 Court Rd, Winthrop, MA 02152, USA

## Abstract

**Background:**

Inhaled short acting β2-agonists (SABA), e.g. albuterol, are used for quick reversal of bronchoconstriction in asthmatics. While SABA are not recommended for maintenance therapy, it is not uncommon to find patients who frequently use SABA over a long period of time and there is a suspicion that long term exposure to SABA could be detrimental to lung function. To test this hypothesis we studied the effect of long-term inhaled albuterol stereoisomers on immediate allergic response (IAR) and airway hyperresponsiveness (AHR) in mouse models of asthma.

**Methods:**

Balb/C mice were sensitized and challenged with ovalbumin (OVA) and then we studied the IAR to inhaled allergen and the AHR to inhaled methacholine. The mice were pretreated with nebulizations of either racemic (RS)-albuterol or the single isomers (S)- and (R)-albuterol twice daily over 7 days prior to harvest.

**Results:**

We found that all forms of albuterol produced a significant increase of IAR measured as respiratory elastance. Similarly, we found that AHR was elevated by albuterol. At the same time a mouse strain that is intrinsically hyperresponsive (A/J mouse) was not affected by the albuterol isomers nor was AHR induced by epithelial disruption with Poly-L-lysine affected by albuterol.

**Conclusions:**

We conclude that long term inhalation treatment with either isomer of albuterol is capable of precipitating IAR and AHR in allergically inflamed airways but not in intrinsically hyperresponsive mice or immunologically naïve mice. Because (S)-albuterol, which lacks affinity for the β2-receptor, did not differ from (R)-albuterol, we speculate that isomer-independent properties of the albuterol molecule, other than β2-agonism, are responsible for the effect on AHR.

## Background

Inhaled short acting beta agonists (SABA) such as albuterol are critical for quick reversal of acute bronchoconstriction in asthmatics. While SABAs are not recommended for maintenance therapy, it is not uncommon for patients to frequently use SABA over an extended period of time and it has been debated whether long term use of SABA is detrimental in asthma [1,2]. β2-agonists are primarily thought to be bronchodilatory drugs acting via relaxation of airway smooth muscle; however, there is also increasing evidence that β2-agonists have other pharmacodynamic effects in the lungs. Terbutaline and formoterol have been shown to inhibit plasma extravasation in inflamed airways of guinea-pigs and rats [3] and formoterol reduced histamine-induced extravasation in humans [4]. Notwithstanding these beneficial effects documented with β2-agonists, they were almost exclusively obtained with racemic compounds and β2-agonists now carry a "black box" warning in many countries because of suspicion that they might worsen asthma if used alone.

Many synthetic drugs, including β2-agonists, exist as racemic mixtures. While the diastereomer has traditionally been considered to be largely inactive, there is accumulating evidence suggesting that isomers without affinity for the β2-receptor may indeed have pharmacological effects of their own [5,6]. In the case of albuterol, the β2-active isomer is (R)-albuterol whereas (S)-albuterol has about 100 times less affinity than does (R)-albuterol for the β2-receptor [7,8]. While there has been a longstanding debate whether the pharmacodynamic effects of diastereomers are of significance or not [9,10], there is also a suspicion that long-term exposure to β2-agonists could be detrimental to lung function [11]. We recently showed that a long acting β2-agonist, salmeterol, worsened respiratory mechanics in a model of allergic asthma [12]. To test the hypothesis that albuterol increases airways hyperresponsiveness in inflamed lungs, we studied the effect of long-term inhaled albuterol stereoisomers on respiratory reactivity in mouse models of asthma, including immediate allergic response (IAR) and allergen induced airways hyperresponsiveness (AHR). Some of the data were previously presented in preliminary form as abstracts at the 2008 and 2009 American Thoracic Society meetings [13,14] and the 2008 IDEA meeting [15].

## Methods

### Animals

Female mice (Balb/C, C57Bl/6 and A/J) were purchased from Jackson Laboratories (Bar Harbor, ME). The mice were housed in an AAALAC and USDA accredited animal facility at the University of Vermont fully equipped for laboratory animal care. The study was approved by the Institutional Animal Care and Use Committee at the University of Vermont.

### Allergen Sensitization

Female mice (Balb/C, 6 - 8 weeks of age) were sensitized and challenged with chicken ovalbumin (OVA). Briefly, on days 0 and 14, animals were injected (100:l, intraperitoneal (i.p.)) with OVA (20 μg) emulsified in 2.25 mg of aluminum hydroxide/magnesium hydroxide.

### Drug inhalation

(R)-, (S)- and (RS)-albuterol were dissolved in phosphate buffered saline (PBS) vehicle and loaded into a Pari nebulizer (6-8 ml). In another study, the Pari nebulizer was reported to produce particles with a mass mean aerodynamic diameter of 2.27 μm with a span of 2.04 μm, with the lung burden of Ova estimated at 10.4 μg per administration [16]. The nebulizer was connected to a multicompartment pie-shaped aerosol chamber where the mice were exposed individually to the aerosol. Nebulizations were delivered early in the morning and late afternoon over 20 minutes. The doses were (R)- (2.5 mg/ml), (S)- (2.5 mg/ml), (RS)- (5 mg/ml) and control PBS vehicle. The doses were chosen to be equipotent on the β2-receptor based on the distribution of (S)- and (R)- in racemic albuterol being 50% of each. The animals were treated for seven consecutive days with the last nebulization 18 hours before readout.

### Intra tracheal administration of Poly-L-lysine

The mice were anesthetized with sodium pentobarbital (90 mg/kg, i.p.) and the trachea cannulated. The mice were then placed supine at about 45°angle and a thin catheter was forwarded through the cannula and 50 μl of the PLL solution followed by about 0.5 ml of air was forcefully injected into the airways. PLL was administrated once 45 minutes before the assessment of AHR with methacholine was started.

### Assessment of the immediate allergic response (IAR)

The mice were immunized i.p. as described above and on days 21-26 were exposed for 30 minutes to an OVA aerosol once daily (1% (w/v) OVA in saline) generated with an ultrasonic nebulizer. Control animals received a saline-only aerosol. The mice were assessed for pulmonary cellular infiltrates, histopathologies, and lung function on day 28. Following about ten minutes of regular ventilation at a positive end-expiratory pressure (PEEP) of 3 cmH_2_O, a standard lung volume history was established by delivering two deep sighs to a pressure limit of 25 cmH_2_O where after two baseline measurements of respiratory input impedance (*Z_rs_*) were obtained. Next, lung mechanics was measured every 10 seconds for 1 minute immediately following inhalation of 5% OVA aerosol (4 separate administrations, one minute challenges with 5 minutes washout in between each challenge) and then once every minute for 20 minutes. OVA aerosol was delivered by temporarily channeling the inspiratory flow from the ventilator through an ultrasonic nebulizer (Beetle Neb, Drive Intl. LLC, NY, particle dimensions 1.5 to 5.7 μm) containing 5% OVA.

### Assessment of airway hyperresponsiveness (AHR)

Balb/C mice were immunized i.p. as described above. On days 21 - 23 they were exposed to 1% OVA aerosol for 30 minutes. Control animals received saline-only aerosol. On day 25 the mice were assessed for airway hyperresponsiveness and pulmonary cellular infiltrates. Lung mechanics was measured on day 25, 48 hr after the last challenge with OVA. Following about ten minutes of regular ventilation at a positive end-expiratory pressure (PEEP) of 3 cmH_2_O, a standard lung volume history was established by delivering two deep sighs to a pressure limit of 25 cmH_2_O. Next, two baseline measurements of respiratory input impedance (*Z_rs_*) were obtained. This was followed by an inhalation of aerosolized control PBS for 40 s, achieved by directing the inspiratory flow from the ventilator through the aerosolization chamber of an ultrasonic nebulizer (Beetle Neb, Drive Intl. LLC, NY). *Z_rs _*was then measured every 10 s for 3 min. Next, two deep sighs were delivered again and two baseline recordings of *Z_rs _*were obtained followed by methacholine inhalation. This was repeated for three incremental doses of methacholine (3.125, 12.5, 50 mg/ml) with measurements as described for PBS.

### Lung mechanics

The mice were anesthetized and cannulated as previously described [17,18]. The cannula was connected to a flexiVent (SCIREQ Inc. Montreal, QC) and ventilated at 200 breaths/minute. *Z_rs _*was determined from a two second broadband perturbation in volume applied by the flexiVent. The data was fitted with the constant phase model [19]. At low frequencies the impedance of the lung is extremely well described by the constant phase model (Eq. 1):(1)

where *R_n _*is the frequency independent Newtonian resistance reflecting that of the conducting airways, *I *is airway gas inertance, *G *characterizes tissue resistance, *H *characterizes tissue stiffness, *i *is the imaginary unit, and *f *is frequency in Hz [19,20].

### Broncho alveolar lavage and cytology

At the end of the protocol the mice were euthanized and the lungs lavaged with 1 ml of phosphate buffered saline. Total cell counts were obtained and the lavage was centrifuged and the supernatant was used for analysis of cytokines (Bio-Plex^® ^Mouse Cyto 23plex), total protein and IgG1. The cell pellet was then re-suspended and cytospin slides prepared for cell differentials using Hematoxylin - Eosin stain.

### Histology

The lung was infused with formalin at 30 cm H_2_O and prepared for histology. Microscopic slides were prepared and stained with Hematoxylin - Eosin to visualize inflammatory cells and morphologic changes. Identification of Clara cells was done by immunohistochemical labeling using an antibody against Clara cell secretory protein (CCSP) (Upstate cell signaling solutions) [21]. For fluorescent labeling of mucin, slides were stained with periodic acid fluorescent Schiff stain (PAFS) to visualize mucus producing cells using fluorescence microscopy. PAFS staining allows for increased specificity of mucin producing cells compared with traditional periodic acid Schiff stain [21]. The slides were scored from 0 (least staining) to 4 (most staining) by three independent persons, masked to the identity of the slides and the scores were then averaged. The scores between persons were not significantly different (p > 0.05).

### Protein analysis

The BALF was analyzed for total protein content using the Bradford protein assay and measured in a plate reader (Bio-Rad).

### IgG1 analysis

The BALF was analyzed for total IgG1 content using ELISA (Pharmingen).

### Statistics

Statistical testing was done with one-way ANOVA with Bonferroni post-hoc test. Statistics were calculated over the entire time-course following each dose of allergen or MCh. Histological scoring was tested with Kruskal-Wallis test and Dunn's multiple comparison post-hoc test. A p < 0.05 was accepted as statistically significant different.

### Experimental design

**Figure 1:**
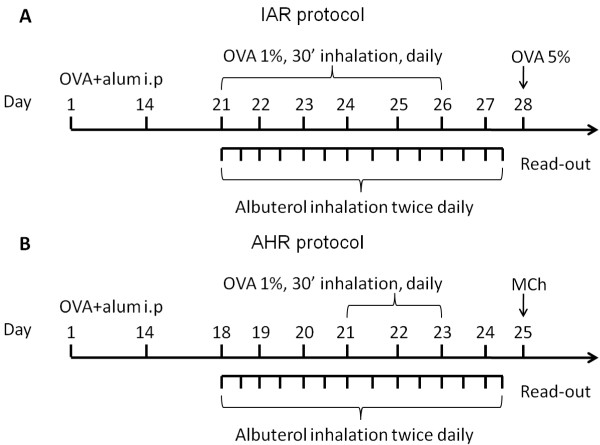
**Timelines of the experiments**. **A**) To measure the immediate allergic airways response (IAR) Balb/C mice were immunized with OVA + Alum i.p. on days 0 and 14. On days 21-26, animals were exposed for 30 minutes to 1% aerosolized OVA; controls received PBS aerosol. Four different groups of mice were treated with nebulized albuterol, (R)- (2.5 mg/ml), (S)- (2.5 mg/ml), (RS)- (5 mg/ml) or control PBS, twice daily for 20 minutes in the morning and in the afternoon on days 21-27. Lung mechanics was measured on day 28 following inhalation of 5% OVA aerosol. **B) **To measure the effect of albuterol on allergen induced AHR, Balb/C mice were immunized with OVA + Alum i.p. on days 0 and 14. On days 21-23 animals were exposed for 30 minutes to 1% aerosolized OVA; controls received PBS aerosol. The mice were treated with nebulized albuterol, (R)- (2.5 mg/ml), (S)- (2.5 mg/ml), (RS)- (5 mg/ml) or control PBS, twice daily for 20 minutes in the morning and in the afternoon on days 18-24. AHR was assessed by measuring *Z_rs _*at increasing doses of inhaled methacholine (MCh).

The study was performed in two steps. The first part of the study focused on elucidating the effects of albuterol isomers on the IAR of the airways (Figure 1A) where as the second part of the study was focused on studying the effect of albuterol isomers on AHR. The latter part of the study was performed in 5 different experiments; naïve C57Bl6 and Balb/C represent mouse strains with normal responsiveness, A/J mice are genetically hyperresponsive, Balb/C mice challenged with Poly-L-lysine and sensitized Balb/C representing allergically inflamed mice that have hyperresponsive airways (Figure 1B).

In the experiment using PLL mice first underwent the drug treatment and then on the day of experiment treated with PLL oropharyngeally and 45 minutes later responsiveness to methacholine was assessed.

## Results

### Immediate Allergic Response (IAR)

#### Physiology

**Figure 2:**
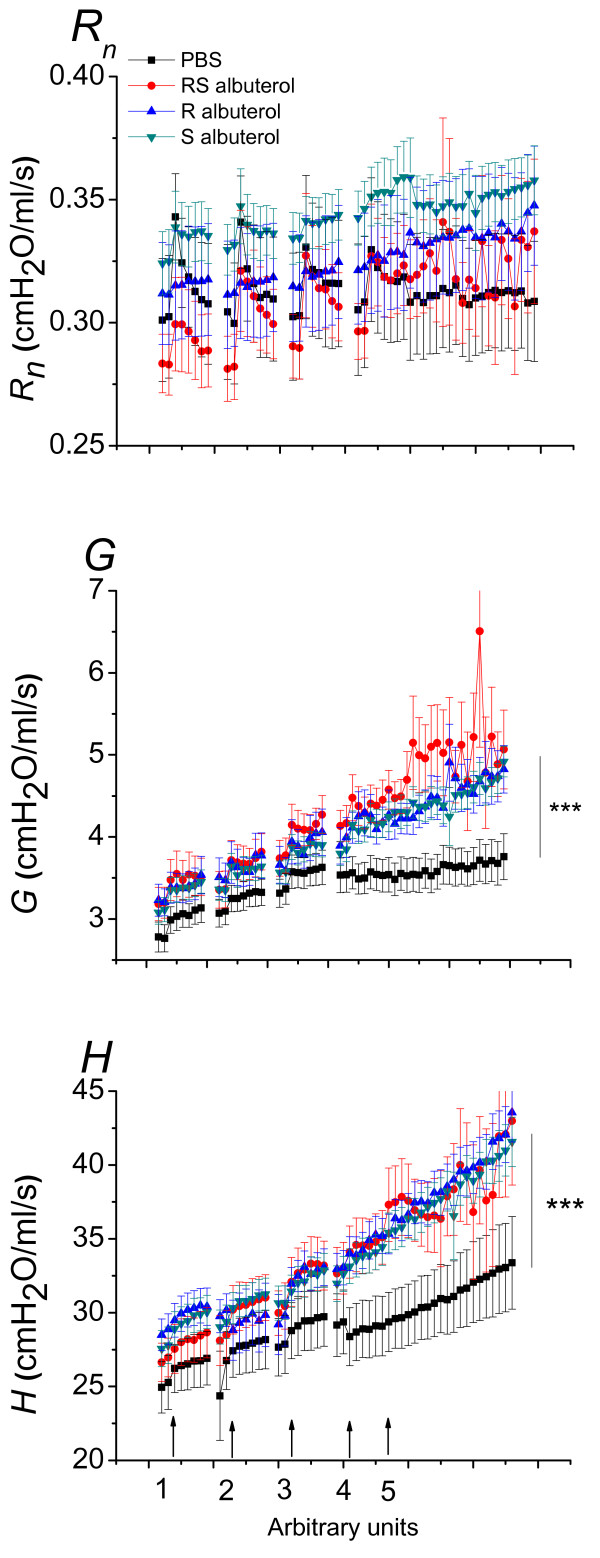
**Effect of albuterol on respiratory mechanics in mice sensitized and challenged with OVA**. Balb/C mice were anesthetized and connected to a flexiVent and then received inhalation challenges of aerosolized OVA. The OVA inhalation was repeated 4 times and the respiratory impedance was measured after each challenge as indicated by arrows and numbers on the X-axis as follows: Following OVA inhalation at indicator 1, 2, 3 and 4 respiratory mechanics was measured every 10 seconds for 1 minute; following OVA inhalation at indicator 5 mechanics was measured once every minute for 20 minutes. Parameters from fitting the constant phase model to input impedance data are shown. *R_n _*is Newtonian resistance of the conducting airways; *H *is lung elastance and *G *is tissue resistance. Animals were treated with (RS)-, (R)- or (S)- albuterol (n = 14, 13 and 15) twice daily for seven days with the last administration 18 hours before experiment. PBS (n = 9) was used as vehicle control. Both *H *and *G *were significantly elevated by (RS)-, (R)- and (S)- albuterol compared with control PBS over the 30 - 60 minutes interval (*** p < 0.001). Changes in *R_n _*were not statistically significant (p > 0.05).

We first investigated the effects of allergen inhalation on respiratory mechanics. Figure 2 shows the respiratory mechanics derived from fitting the constant phase model of the respiratory system to *Z_rs _*data in OVA challenged mice. OVA inhalations produced small but reproducible increases in *R_n _*in all groups except the group that received (R)-albuterol. There were, however, no statistical differences between groups (p > 0.05). After the fourth OVA exposure, lung mechanics were measured every minute for 20 minutes. *R_n _*did not change significantly over the 20 minute period, with either treatment, however, *G *and *H *increased significantly over control (p < 0.001) in the mice treated with (RS)-, (S)- and (R)-albuterol.

#### Cytology

**Figure 3:**
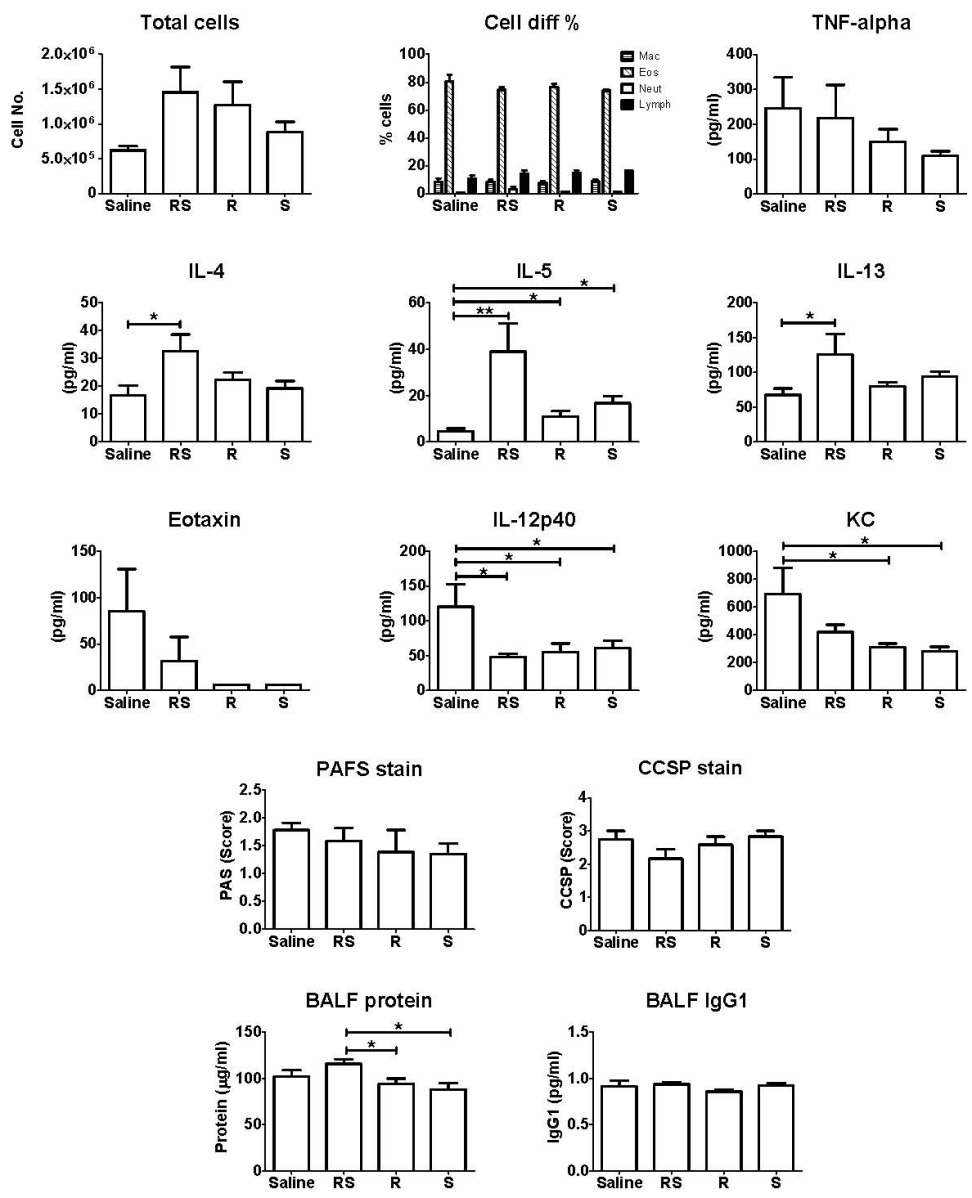
**Cell differentials, cytokine titers, plasma indicators from BALF and histology scores**. Following euthanasia, lungs were lavaged with 1 ml of PBS, and cells were counted, and cytospin slides were stained with H&E (n = 5 in each group). No statistically significant differences were found between treatment groups (p > 0.05). Cytokine concentrations were measured from the BALF supernatant using Bio-Plex^®^. * p < 0.05, ** p < 0.01, (n = 10 in each group). Results of scoring of PAFS stained histological sections of lungs; no statistical difference was found between groups. Results of scoring of CCSP staining; no statistical difference was found between groups (n = 5 in each group). Total BALF protein was significantly increased in mice treated with (RS)-albuterol compared with (R)- and (S)-albuterol (* p < 0.05) whereas IgG1 in BALF was not affected by either treatment (n = 10 - 18 in each group).

After euthanasia the lungs were lavaged *in situ *with PBS and the cellular inflammation was assessed. Figure 3 shows the result from counts of cells in BALF from mice challenged with OVA. The cell number was increased in all treated mice. The cytology was dominated by eosinophils but neither treatment had any significant effect on the relative cell differentials (% cell numbers).

#### Cytokines

The sensitization and challenge protocol we use typically produces a Th2 dominated cytokine profile; hence we wanted to confirm this in this experiment. Figure 3 shows cytokine levels obtained from the Bio-Plex assay. We found that KC and IL-12(p40) analyzed in bronchoalveolar lavage were significantly decreased by treatment with (RS)-, (R)- and (S)- albuterol (p < 0.05). IL-5, IL-4 and IL-13 were significantly elevated over saline control only by (RS)-albuterol (p < 0.05), commensurate with the expected Th2 profile.

#### Histology

Mucus expression has been shown to be linked to AHR [22] but it is not known if mucus expression is increased following an IAR or if it would be affected by albuterol. As shown in Figure 3 we determined the expression of mucus by scoring PAFS stained slides of lungs obtained from mice that were treated with either isomers of albuterol or control saline post OVA challenge. The staining of mucin was not different between the groups. Similarly we found that the immunomodulatory and anti-inflammatory CCSP was not affected by albuterol treatment.

#### Protein and IgG1

It has been shown that various challenges to the airway mucosa can induce plasma extravasation [23] and it has been suggested that components of the extravasate can contribute to AHR [24]. We used IgG1 and total protein content of the BALF as indicators of plasma leakage. Figure 3 shows the results from the protein and IgG1 analysis in BALF. The total protein content of the BALF was significantly increased in (RS)-albuterol treated mice compared with (R)- and (S)- treated, however, there was no difference compared with the control group. IgG1 was measured as an indicator of plasma leakage. There was, however, no difference in BALF IgG1 levels between treatments suggesting that no significant exudation took place.

### Airways Hyperresponsiveness (AHR)

#### Physiology

**Figure 4:**
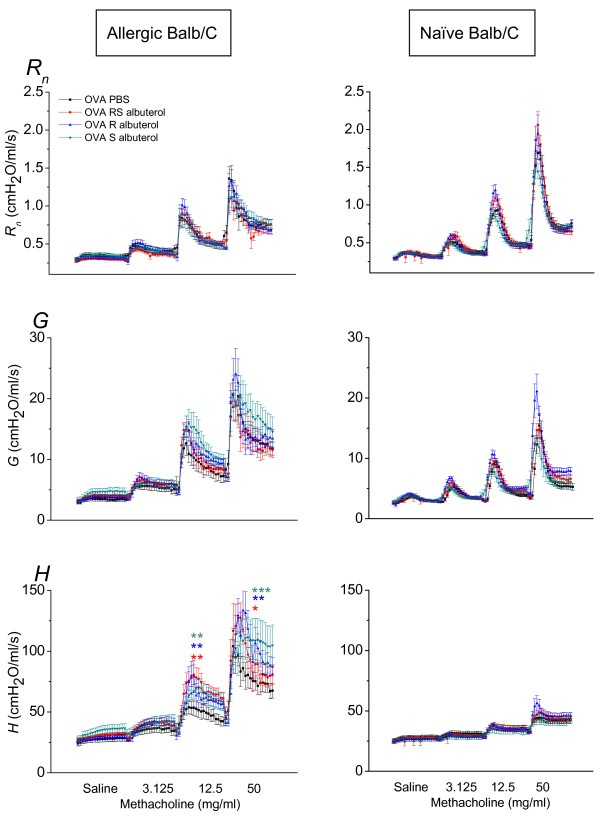
**Effect of albuterol on AHR in allergic mice**. Respiratory mechanics time course following methacholine challenge. Parameters from fitting the constant phase model to input impedance data; *R_n _*is Newtonian resistance of the conducting airways; *H *is lung elastance and *G *is tissue resistance. Left column: Allergic Balb/C mice; AHR measured as dose-response time-course to increasing doses of methacholine inhalation in OVA sensitized Balb/C mice. Animals were treated with (RS)-, (R)- or (S)- albuterol (n = 11, 12 and 10) twice daily for seven days with the last administration 18 hours before experiment. PBS (n = 10) was used as vehicle control. *H *was significantly elevated over PBS control at the 12.5 and 50 mg/ml doses of methacholine by (RS)-, (R)- and (S)- albuterol. * p < 0.05, ** p < 0.01 and *** p < 0.001. Right column: Naïve Balb/C mice; AHR assessment in naïve Balb/C mice. Animals were treated with (RS)-, (R)-, (S)- albuterol or control PBS, n = 8 per group.

**Figure 5:**
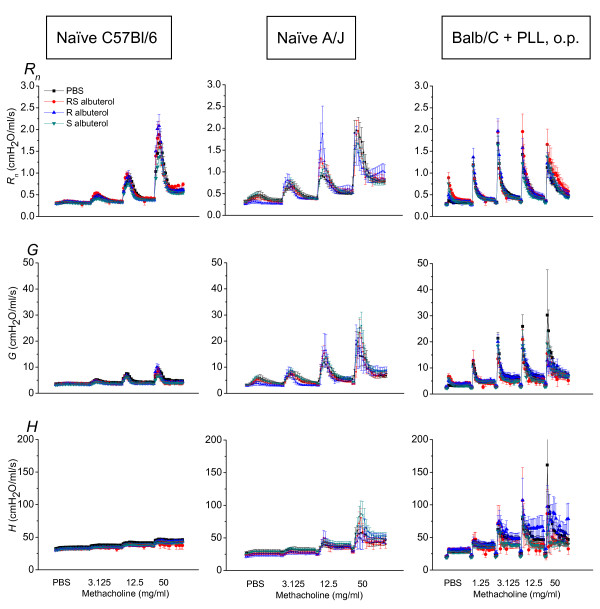
**Effect of albuterol on AHR in non-allergic mice**. Respiratory mechanics time course following methacholine challenge. Animals were treated with albuterol twice daily for seven days with the last administration 18 hours before experiment. Parameters from fitting the constant phase model to input impedance data; *R_n _*is Newtonian resistance of the conducting airways; *H *is lung elastance and *G *is tissue resistance. Left column: AHR assessment in naïve C57Bl/6 mice, treated with (RS)-, (R)-, (S)- albuterol or control PBS (n = 8, 6, 7 and 8). Middle column: AHR assessment in naïve A/J mice. Animals were treated with (RS)-, (R)-, (S)- albuterol or control PBS (n = 8, 5, 7 and 7). Right column: AHR assessment in Balb/C mice pretreated oropharyngeally with Poly-L-lysine (PLL) (50 μg in 50 μl PBS). PLL was administered once daily for 4 consecutive days before the assessment of respiratory mechanics with methacholine. Animals were treated with (RS)-, (R)-, (S)- albuterol or control PBS (n = 6, 6, 7 and 8).

AHR is a hallmark of allergically inflamed airways, thus we next studied the effect of (RS)-, (S)- or (R)- albuterol treatment on AHR in allergically sensitized and challenged mice. This was done measuring *Z_rs _*at increasing doses of methacholine. Figure 4 shows the respiratory mechanics dose-response to incremental methacholine inhalations in allergic Balb/C mice. Treatment with either (RS)-, (S)- or (R)-albuterol had no significant effect on the increase in *R_n _*or *G*. All treatments did, however, significantly increase the response in *H*, commensurate with increased lung stiffness due likely to airway closure [18]. This finding then prompted us to investigate if albuterol would affect the airways responsiveness of naïve mice. We studied this in three different strains of mice previously shown to have different degrees of responsiveness to methacholine. Other studies have shown the order of sensitivity to methacholine to be A/J > Balb/C > C57Bl/6 [25-27], with A/J often considered to be genetically hyperresponsive. We found that naïve mice of all of these strains were unaffected by either (RS)-, (S)- or (R)- albuterol treatment to a significant degree (Figures 4 and 5).

Another predisposition for AHR could be epithelial injury, as is frequently seen in asthma. The epithelial lining of the airways is damaged by inflammatory processes and it has been suggested that desquamation and denudation of the epithelium are significant features of asthma [28]. Although the causes of epithelial injury can be multiple, one source that is likely to be important is the release of cationic proteins from eosinophils. When eosinophils degranulate they release major basic protein (MBP), a cationic protein that may injure the epithelium [29]. We have previously shown that PLL increase AHR via epithelial disruption and that this manifests in the conducting airways suggesting that access to the smooth muscle was facilitated by PLL [30]. Thus, we wanted to determine whether increasing the AHR with PLL would be affected by albuterol. Figure 5 shows the respiratory mechanics from Balb/C mice challenged oropharyngeally with PLL. Neither pretreatment with (RS)-, (S)- or (R)- albuterol had any effect on the methacholine dose-response following PLL.

## Discussion

We have performed a detailed assessment of the effects of racemic albuterol as well as its separate isomers on the respiratory phenotype. In particular we focused on the effects of albuterol isomers on allergen and methacholine perturbed respiratory mechanics following an extended period of pretreatment with inhaled albuterol. We were interested to investigate if albuterol might induce effects that would persist beyond termination of administration, therefore the study was designed in such a manner that drugs were delivered twice daily over seven days and then stopped 18 hours before analysis. With this approach, the drug had time to wash out and we were studying only the sequelae of the treatment and not the direct effect of the drug, such as bronchial relaxation. First, we studied whether albuterol affects allergen induced responses in the lung. We found that the IAR in terms of *H *and *G *were increased. With this piece of information, we then speculated that AHR might also be affected. Hence, we studied the effect of albuterol on allergen-induced AHR and discovered that AHR in terms of *H *was elevated by treatment with (RS)-, (S)- and (R)- albuterol. Finally we tested whether the AHR could be due to epithelial disruption or effects on the smooth muscle and found that neither could explain the increase in AHR caused by extended albuterol treatment.

We triggered the IAR by administering nebulized OVA to allergic mice and then immediately started tracking the respiratory mechanics. We expected the OVA to trigger a constriction of airway smooth muscle that would be seen as an increase in *R_n_*. The responses in *R_n _*elicited by OVA were generally small, but repeatable and seemed to be inhibited by (R)-albuterol, although not to a statistically significant extent (Figure 2). If we compare the amplitude of the responses in *R_n _*following an OVA challenge with the response seen in lungs challenged with methacholine in Figures 4 and 5, we conclude that the airway constriction elicited by inhaled allergen is very small and probably does not carry much biological significance in the airways of mice. The increase in *H *and *G *following the allergen challenge, on the other hand, were much more pronounced over time in the presence of (RS)-, (S)- or (R)- albuterol. These observations illustrate that mice are capable of generating a smooth muscle response in the conducting airways when exposed to allergen, however, the muscle response was small and the result demonstrate that the conducting airways are probably not the location in which most of the activity of the allergen takes place. Instead, the allergen induced effects in the lung periphery (*H *and *G*) were augmented with (RS)-, (S)- or (R)- albuterol likely due to closure of peripheral airways [18].

Inhalation of allergen is a common trigger of asthma and instigates an immediate release of mediators from mast cells that have the capacity to activate a number of pathways that lead to lung inflammation and AHR [31]. Some of the mast cell mediators, e.g. histamine and serotonin, have the capacity to stimulate smooth muscles to contract, whereas other mediators are involved in the cascade that leads to overt inflammation, including recruitment of leucocytes, plasma leakage and eventually AHR [32,33]. The immediate response to an allergen challenge is usually manifest as a bronchoconstriction of the conducting airways leading to a reduction of airflow and shortness of breath [33]. Typically, this IAR can be successfully treated with inhaled bronchodilators such as albuterol. The notion that β-agonists can cause a decline in lung function is neither new nor is it limited to observations in animal models. It was noted in a year-long study that asthmatic patients treated as needed with racemic fenoterol resulted in more exacerbations, a significant decline in baseline lung function, and an increase in airway responsiveness to methacholine, but did not alter bronchodilator responsiveness [34]. As indicated by our results, one explanation to the deteriorating lung function in patients could be that the albuterol treatment increased the propensity for airway closure following allergen challenge.

We next addressed the cause of airway closure exacerbated by prolonged albuterol treatment by exploring two alternative hypotheses. The first is that increased mucus production from the epithelial cells is promoted by albuterol treatment. The second is that albuterol treatment increases plasma leakage into the lung. We studied the mucus producing epithelial cells in a semi-quantitative manner and found that the score of PAFS positive cells was not augmented by any treatment. We then focused on quantification of extravasation in the BALF and used IgG1 and total protein in BALF as indicators of plasma extravasation. The increase in total protein in the (RS)-albuterol treated mice was small but significant compared with (R)- and (S)-albuterol treated mice, suggesting that (R)- and (S)-albuterol, which otherwise had no significant effect on plasma extravasation on their own, may have mild detrimental effects on plasma extravasation when administered simultaneously as a racemic mixture. IgG1 extravasation into the lung, on the other hand, was not affected by albuterol. A recent study from our group demonstrated that AHR induced by acute acid aspiration correlates with BALF protein, whereas this correlation was lost over time, possibly due to healing of the acid induced epithelial injury [35]. The techniques we used to study extravasation herein do not directly measure plasma leakage, hence, we are unable to completely rule out the possibility that plasma leakage did occur. Notwithstanding this uncertainty, our data do not support plasma extravasation as a mechanism for why the isomers of albuterol and the racemic mixture produced similar degrees of airway closure.

We performed an extensive analysis of BALF cytokines one hour post allergen challenge. While the concentrations of most cytokines did not change and the titers were generally low, we found that IL-4, IL-5 and IL-13 were significantly increased in mice treated with (RS)-albuterol. These cytokines are conventionally considered as Th2 cytokines and thought to promote the asthma phenotype [36]. Chronic administration of various racemic β2-agonists have been shown to induce increased production of pro-inflammatory IL-13 in Th2 cells from asthmatic patients *in vitro*, which was suggested to be independent of the isomer of albuterol[37]. In this context, it is interesting to note that in our study the single isomer (R)-albuterol did not significantly induce inflammatory cytokines. However, when (S)-albuterol was present in the form of (RS)-albuterol, the picture changed in the direction of more Th2 cytokines being produced. The significant decreases in IL-12p40 in the BALF from mice receiving (RS)-albuterol may partially explain the observed increases in Th2 cytokines from these same mice, as IL-12p40 acts as a negative regulator of IL-12p70 signaling [38], which itself functions to promote Th1 responses that antagonize Th2. The increase in Th2 cytokines did not seem to affect respiratory mechanics, as we did not measure any difference between (RS)-albuterol and the pure isomers when the mice were challenged with allergen. Studies *in vitro *have shown that (S)-albuterol may activate mast cells and enhance release of histamine and IL-4 [39], which could adversely affect patients.

The total cell number present in lavageable airspaces appeared increased in all treatment groups although not statistically significant (Figure 3) and the cell differentials revealed that the inflammation was dominated by eosinophils.

A significant problem in asthma is the hyperresponsiveness to various inhaled stimuli [40,41]. Testing patients for hyperresponsiveness helps in setting the diagnosis of asthma. As it has been suggested that extensive β2-agonist treatment might contribute to the development of hyperresponsiveness, we designed experiments to address this issue *in vivo *in different animal models. We found that pretreatment with either compound had an effect on methacholine induced hyperresponsiveness in allergic mice (Figure 4). This was evidenced by a significant increase in *H *commensurate with an increase in lung de-recruitment [18]. From these data, we draw the conclusion that β-receptor independent properties of albuterol appear to augment the AHR in allergic mice. We also found that (S)-albuterol did not affect *H *neither in a strain known to be genetically hyperresponsive (A/J (Figure 5) nor in normal responsive animals (non-allergic Balb/C and C57Bl/6 (Figure 4, 5)). A/J mice exhibit AHR as an increase in *R_n_*, which in turn depends on the airway smooth muscle having a higher shortening velocity in the A/J compared to that of most other mouse strains [26,42]. Since AHR was not affected by albuterol in A/J mice (Figure 5), this suggests that the AHR increase in OVA sensitized mice was probably not due to effects on the airway smooth muscle. Thus, it appears that preexisting lung inflammation is necessary for albuterol to cause further negative effects on the hyperresponsiveness of the respiratory system. Since each of the isomers of albuterol, as well as the racemic mixture, increased AHR, the mechanism must be β-receptor independent.

When comparing the results obtained with IAR and AHR we noticed a qualitative difference in that inhaled OVA (Figure 2) generated an increase in both *G *and *H*, whereas inhaled methacholine (Figure 4) produced only an increase in *H*. We speculate that these differences are explained by the different modes of action of methacholine and OVA. Methacholine stimulates airway smooth muscle directly via muscarinic receptors, accounting for the effect on *R_n_*. Methacholine is also a secretagogue with the capacity to trigger epithelial cells to expel mucus [43] which might account for airway closure and the increase in *H*. OVA, on the other hand, acts more indirectly via intermediary resident and inflammatory leukocytes (i.e. mast cells) [32] that conceivably could trigger both mucus secretion and alterations in the visco-elastic properties of the lung, thereby leading to a more complex response including both *G *and *H*.

It is, of course, difficult to compare clinical asthma with our mouse model particularly since we used a long-term treatment protocol followed by a wash-out period. While only a few clinical studies with (S)-albuterol have been performed the results have been mixed. Two crossover trials failed to detect any increase in AHR with a single dose of 100 μg (S)-albuterol [44,45], whereas another study detected an increase in AHR, albeit after a much higher single dose of (S)-albuterol, (5 mg) [46]. Taken together, this might suggest that either high doses or sustained treatment with albuterol is needed to reveal any adverse effects on AHR.

We administered a model cationic protein, PLL, that mimics MBP from eosinophils, which has been shown to induce increased permeabilization of the epithelial lining [47] with subsequent hyperresponsiveness to inhaled methacholine, which in turn is probably due to increased epithelial permeability primarily affecting the conducting airways [30,48]. It has also been shown that salmeterol prevents compromise of the airway epithelial barrier when histamine-1 receptor or Protease Activated Receptor-2 were activated in primary airway epithelium [49]. We used PLL expecting that it would reveal effects of the long-term treatment with albuterol isomers on the smooth muscle. The hypothesis was that the smooth muscle would normally be protected by an intact epithelium disguising the effect of methacholine. We found that PLL induced a robust response to methacholine comparable to what has been shown before by our group [50], however, pretreatment with albuterol did not affect the response in any manner. Since albuterol did not affect AHR in Poly-L-lysine treated mice (Figure 5) nor in non-allergic mice (Figure 4 and 5), we conclude that the AHR in OVA allergic mice was probably not due to changes in epithelial permeability.

## Conclusion

In summary, we have determined the effects of chronic (R)-, (S)- and (RS)-albuterol treatment on IAR and AHR in mice. We found that all three drugs were equally effective in causing peripheral airway closure following an allergen challenge. The closure was not caused by mucus production or by increased plasma extravasation. All three compounds also increased the AHR to a similar degree. The expression of Th2 cytokines was somewhat elevated in mice treated with (RS)-albuterol; however, this did not lead to a unique phenotype. The effects of chronic albuterol treatment were not attributable to epithelial disruption because albuterol was not affected by PLL instillation. In addition, the smooth muscle did not seem to be involved because AHR in A/J mice was not affected by albuterol treatment. These observations also suggest that the airways are not negatively affected by albuterol but rather that the periphery of the lung is sensitive to adverse effects by albuterol. Interestingly, our data demonstrate that pulmonary inflammation seems to be a prerequisite for albuterol to produce increased responses to either allergen or MCh because naïve mice did not change their response following albuterol treatment. Finally, we are left with the notion that the individual enantiomers and racemic albuterol share the same ability to affect the lung phenotype whether induced by allergen inhalation or broncho constriction with MCh and that this ability is not related to the β2-receptor but is due to some other property of the albuterol molecule that is unrelated to its steric configuration.

## Competing interests

LKAL received investigator initiated grants from Sepracor Inc. ($195,169 and $197,658) and received $9,000 in consultancy remuneration from Sepracor Inc. MEP received an investigator initiated grant from Sepracor Inc. ($100,000) and received $9,000 in consultancy remuneration from Sepracor Inc. LMB was an employee with Sepracor Inc. at the time of the study. CGI received an investigator initiated grant from Sepracor Inc. ($30,000) and $4,000 in consultancy remuneration from Sepracor Inc. LMR, PER, MW, SA, JHTB declare that they have no competing interests.

## Authors' contributions

LKAL designed the study and supervised and participated in the experiments in their entirety and data analysis, he also wrote the manuscript. LMR, MW and SA performed the *in vivo *experiments, did cell differentials, histological staining, protein analysis, and assisted with data analysis. EPR did the analysis of the histology and assisted in manuscript writing. MEP did the Bio-Plex^® ^cytokine analysis and assisted in data interpretation and manuscript writing. LMB participated in the study design, data interpretation and manuscript writing. JHTB assisted with data interpretation and manuscript writing. CGI was involved in the study design and in the preparation of the manuscript. All authors have read and approved the final manuscript.
